# Retinal neurodegeneration in patients with type 1 diabetes mellitus: the role of glycemic variability

**DOI:** 10.1007/s00592-017-0971-4

**Published:** 2017-02-25

**Authors:** Fabiana Picconi, Mariacristina Parravano, Dorina Ylli, Patrizio Pasqualetti, Sara Coluzzi, Ilaria Giordani, Ilaria Malandrucco, Davide Lauro, Fabio Scarinci, Paola Giorno, Monica Varano, Simona Frontoni

**Affiliations:** 1 Department of Systems Medicine, University of Rome Tor Vergata, Rome, Italy; 2 Unit of Endocrinology, Diabetes and Metabolism, S. Giovanni Calibita Fatebenefratelli Hospital, Rome, Italy; 3 IRCCS-G.B. Bietti Foundation, Rome, Italy; 4 Service of Medical Statistics and Information Technology, Fatebenefratelli Foundation for Health Research and Education, AFaR Division, Rome, Italy

**Keywords:** Retinal neurodegeneration, Glycemic variability, Type 1 diabetes mellitus

## Abstract

**Aims:**

Recent studies have identified neuroretinal abnormalities in persons affected by diabetes mellitus, before the onset of microvascular alterations. However, the role of glycemic variability (GV) on early retinal neurodegeneration is still not clarified.

**Methods:**

To explore the relationship between glycemic control and neuroretinal characteristics, 37 persons with Type 1 diabetes mellitus (Type 1 DM) divided into two groups with no signs (noRD) and with mild non-proliferative diabetic retinopathy (NPDR) compared to 13 healthy control participants (*C*) were recruited. All persons underwent an optical coherence tomography with automatic segmentation of all neuroretinal layers. Measurements of mean of nasal (*N*)/temporal (*T*)/superior (*S*)/inferior (*I*) macular quadrants for individual layer were also calculated. Metabolic control was evaluated by glycated hemoglobin (HbA1c), and indexes of GV were calculated from continuous glucose monitoring.

**Results:**

The difference among the three groups in terms of RNFL thickness was significantly dependent on quadrant (*F*(6;132) = 2.315; *p* = 0.037). This interaction was due to a specific difference in RNFL-N thickness, where both Type 1 DM groups showed a similar reduction versus C (−3.9 for noDR and −4.9 for NPDR), without any relevant difference between them (−1.0). Inner nuclear layer (INL) was increased in all quadrants in the two Type 1 DM groups compared to *C* (mean difference = 7.73; 95% CI: 0.32–15.14, *p* = 0.043; mean difference = 7.74; 95% CI: 0.33–15.15, *p* = 0.043, respectively). A negative correlation between RNFL-N and low blood glucose index (*r* = −0.382, *p* = 0.034) and positive correlation between INL and continuous overall net glycemic action −1, −2, −4 h (*r* = 0.40, *p* = 0.025; *r* = 0.39, *p* = 0.031; *r* = 0.41, *p* = 0.021, respectively) were observed in Type 1 DM patients. The triglycerides were positively and significantly correlated to INL (*r* = 0.48, *p* = 0.011), in Type 1 DM subjects. GV and triglycerides resulted both independent predictors of increased INL thickness. No correlation was found with HbA1c.

**Conclusions:**

Early structural damage of neuroretina in persons with Type 1 DM patients is related to glucose fluctuations. GV should be addressed, even in the presence of a good metabolic control.

## Introduction

As the worldwide prevalence of diabetes mellitus continues to raise, diabetic retinopathy represents a leading cause of vision loss in many developed countries [1]. The number of people at risk of developing vision loss from diabetes is predicted to double over the next 30 years [2], so it is crucial to develop better means to prevent, identify and treat retinopathy in its earliest stages.

In a recent review, Cheung et al. [3] included exposure to persistent hyperglycemia, longstanding diabetes, altered blood pressure control and certain ethnic origins among the well-established risk factors for DR. Moreover, a single-nucleotide polymorphism Rs2910164 was significantly associated with DR in patients affected by Type 1 and Type 2 diabetes mellitus (Type 1 DM; Type 2 DM) [4]. In Asian populations, BMI levels were inversely associated with DR [5], which may be due to better *β* cell function in overweight patients [6].

Hyperglycemia instigates the cascade of events that finally leads to development of diabetic retinopathy. However, the role of glycemic control, expressed as HbA1c, on the development of RD is still not fully clarified. Recently, daily glycemic variability (GV) has been proposed as a major contributor to the development of diabetes macrovascular and cerebrovascular complications [7–11]. However, the association between GV and diabetes microvascular complications has yielded inconsistent findings. Recent evidence has emphasized the role of neuroretina in the interactions between neural alterations and microvascular abnormalities [12], and the relationship between GV and retinal neurodegeneration, in the early stages of RD, has been only very recently investigated [13].

The purpose of the present study was to investigate the relative impact of overall glycemic load and of GV on structural changes of neurosensory retina, in persons with Type 1 DM, with no signs (noDR) or mild non-proliferative diabetic retinopathy (NPDR) and without microvascular complications, i.e., absence of peripheral neuropathy and microalbuminuria.

## Methods

From the Unit of Endocrinology, Diabetes and Metabolism, S. Giovanni Calibita Fatebenefratelli Hospital of Rome, we recruited 37 patients with Type 1 DM, divided into two groups: noRD group (19 patients) and NPDR group (18 patients).Thirteen healthy participants, without history of ocular disease, no family history of glaucoma or any relevant systemic disease, were enrolled as control group (*C*), from the medical staff of Department of Ophthalmology, G.B. Bietti Eye Foundation-IRCCS, Rome.

Inclusion criteria for the Type 1 DM patients were: (1) documented diagnosis of Type 1 DM, according to ADA criteria [14]; (2) age between 18 and 75 years; (3) treated with continuous subcutaneous insulin infusion or with multiple daily insulin injections; (4) no signs of retinal vasculopathy or NPDR. Exclusion criteria were: (1) symptomatic diabetic polyneuropathy affecting the lower extremities with positive sensory symptoms such as pain, burning, paresthesia or prickling; (2) abnormal amplitude latency or conduction velocity in motor nerve (either tibial or deep peroneal) and/or in the sural nerve; (3) a Michigan Diabetes Neuropathy Instruments [15] equal to or greater than 2 points; (4) microalbuminuria (urinary albumin/creatinine ratio >30 mg/g); (5) spherical refractive error >±6 diopters, astigmatism (cyl) >±3 diopters, active or past retinal pathologies, diagnosis of glaucoma or ocular hypertension, opacities of optical media that could influence functional and structural retinal testing; (6) history of ocular surgery. Informed consent was obtained from all individual participants included in the study.

### Study design

All subjects underwent a general medical examination and anthropometric parameters. After an overnight fast, blood and urine samples were obtained for the determination of laboratory measurements. Each person underwent a complete ophthalmic examination, with determination of best corrected visual acuity, anterior segment examination, spectral domain optical coherence tomography (SD-OCT) and fundus photography. All Type 1 DM persons were subjected to 72-h continuous glucose monitoring (CGM).

### Ophthalmic Assessment

Color stereoscopic fundus photographs were taken after an adequate dilatation by a trained photographer (TRC-50×; Topcon Instrument Corp., Tokyo, Japan). Diabetic retinopathy was graded as noDR and as NPDR by two independent graders experienced in grading DR (MP, FS).

OCT-SD scanning was performed using Heidelberg Spectralis version 1.9.10.0 (Heidelberg Engineering, Heidelberg Germany) with the IR and OCT30 degrees ART examination procedure. A standard imaging protocol used consisted of 20° × 20° volume scans of the macula area with 49 B-scans, with a mean of 16 automatic real-time images (ART) per scan in high-resolution mode. Clinical study-certified investigators performed all images.

Macular measurements were performed using the inbuilt Spectralis mapping software, Heidelberg Eye Explorer (version 6.0c). The Spectralis segmentation software [16] was used to obtain individual retinal layer thickness measurements including: overall retinal thickness (RT), retinal nerve fiber layer (RNFL), ganglion cell layer (GCL), inner plexiform layer (IPL), inner nuclear layer (INL), outer plexiform layer (OPL), outer nuclear layer (ONL), retinal pigment epithelium (RPE), inner retinal layer (IRL) and photoreceptor layer (PR) thickness. The Spectralis mapping software generates automated measures of retinal thickness based on analyses of the central and inner 1000, 3000 and 6000 microns subfield as defined by the Early Treatment Diabetic Retinopathy Study [17]. Measurements of mean of subfoveal, inner and outer nasal (*N*)/temporal (*T*)/superior (*S*)/inferior (*I*) quadrants for individual layer were also calculated. No manual adjustments to B-scan retinal layer segmentation were used prior to measurements.

### Assessment of GV indexes

After an overnight fast, the Type 1 DM group underwent 72-h CGM with the Ipro2 System (Medtronic, Northridge, CA), and a subcutaneous sensor (Enlite; Medtronic, Northridge, CA) for CGM was applied on the same day of SD-OCT analysis. From CGM data, the following indexes of GV were calculated [18]: standard deviation (SD); mean amplitude of glucose excursion (MAGE); *J*-index; mean absolute glucose (MAG); continuous overall net glycemic action (CONGA-1, -2 and -4); low blood glucose (LBGI) and high blood glucose index (HBGI); *M* value.

### Laboratory measurements

Plasma glucose concentrations were measured by the hexokinase method (Modular P Analyzer; Roche). The intra-assay coefficients of variation (CV) were 1.1% and inter-assay CV was 1.9%. The sensitivity of the method was 2 mg/dL (0.11 mmol/L). HbA_1c_ was analyzed by high-performance liquid chromatography (VARIANT 2; BioRad Laboratories, Munich, Germany), with intra- and inter-assay CV of 0.46–0.77 and 0.69–0.91%, respectively.

Plasma total cholesterol, high-density lipoprotein (HDL chol) cholesterol and low-density lipoprotein cholesterol (LDL chol) were analyzed with a colorimetric enzymatic method (CHOD-PAP; Roche Diagnostics). The intra-assay CV was 1%, and the inter-assay CV was 2.7%. The sensitivity of the method was 0.08 mmol/L. Plasma triglycerides were analyzed with a colorimetric enzymatic method (GPO-PAP; Roche Diagnostics). The intra-assay CV was 1.5%, and the inter-assay CV was 2.4%. The sensitivity of the method was 0.05 mmol/L.

Urinary albumin was determined by the Tina-quant immunoturbidimetric assay (Cobas; Roche Diagnostic, Indianapolis, IN) and urinary creatinine by enzymatic colorimetric test (Beckmann Coulter, California, USA).

C underwent an oral glucose tolerance test, to exclude diabetes and impaired glucose tolerance.

### Statistical analysis

Differences among noDR, NPDR and C groups were assessed by means of general linear model, taking into account the eventual variance heterogeneity. A measure (eta-squared) of the standardized effect size (SES) was provided allowing to check whether the absence of statistically significant effects could be attributed to the small samples size.

Correlations among interval variables were measured through Pearson’s index, after appropriate log-transformation when necessary. In order to evaluate the adjusted effects of multiple variables on retinal thickness layers, multivariable regression analysis was applied.

## Results

Clinical and laboratory characteristics of noDR and NPDR groups of diabetic patients and of C group are reported in Table 1. The three groups were not different, except for age, body mass index (BMI), fasting glucose and HDL chol. C group was younger than NPDR, had lower BMI than NPDR and, as expected, lower plasma glucose concentration than both Type 1 DM groups. The two groups of diabetic patients were comparable for sex, HbA1c, anthropometric characteristics and blood pressure values. However, age and diabetes duration were significantly higher in NPDR, as expected, and HDL chol significantly lower in the NPDR subgroup.

**Table 1:** Clinical and laboratory characteristics of the diabetic group noDR and NPDR compared to C group, mean (SD)

	C *n* = 13	NoDR *n* = 19	NPDR *n* = 18	Test degrees of freedom *p* value
Gender (M/W)	5/8	9/10	10/8	*χ* ^2^ = 0.889
				*df* = 2
				*p* = 0.641
Age (years)	35.8 (6.1)	38.2 (10.9)	44.9 (9.1)^b,c^	*F* = −3.934
				*df* = 2.47
				*p* = 0.027
				*η* ^2^ = 0.15
Diabetes duration (years)	–	15.8 (10.4)	22.4 (10.3)^c^	*t* = −2.269^*a*^
				*df* = 35
				*p* = 0.030
				*η* ^2^ = 0.13
BMI (kg/m^2^)	22.6 (2.0)	24.4 (3.6)	25.7 (3.1)^b^	*F* = −3.606
				*df* = 2.47
				*p* = 0.035
				*η* ^2^ = 0.14
Glycemia (mmol/L)	4.8 (0.6)	8.2 (1.6)^b^	8.8 (2.0)^b^	*F* = −28.008
				*df* = 2.47
				*p* < 0.001
				*η* ^2^ = 0.55
HbA1c (%; mmol/mol)	–	7.77 (1.01) 61.33 (11.07)	8.08 (1.11) 64.79 (12.17)	*t* = −0.283
				*df* = 36
				*p* = 0.779
				*η* ^2^ = 0.02
Tot chol (mmol/L)	4.0 (0.6)	4.5 (1.1)	4.6(1.3)	*F* = −1.075
				*df* = 2.47
				*p* = 0.350
				*η* ^2^ = 0.04
HDL chol (mmol/L)	1.7 (0.1)	1.8 (0.3)	1.5 (0.3)^c^	*F* = −6.012
				*df* = 2.47
				*p* = 0.005
				*η* ^2^ = 0.20
LDL chol (mmol/L)	2.0 (0.6)	2.4 (0.9)	2.6 (1.0)	*F* = −1.609
				*df* = 2.47
				*p* = 0.211
				*η* ^2^ = 0.06
Trigl (mmol/L)	0.8 (0.1)	0.8 (0.3)	1.0 (0.5)	*F* = −1.853
				*df* = 2.47
				*p* = 0.168
				*η* ^2^ = 0.07
Microalb/creat (mg/g)	–	7.1 (6.3)	5.8 (2.7)	*t* = −0.421^a^
				*df* = 15.4
				*p* = 0.679
				*η* ^2^ = 0.01
SBP (mmHg)	–	122.4 (18.5)	122.2 (31.5)	*t* = 0.018
				*df* = 36
				*p* = 0.986
				*η* ^2^ < 0.01
DBP (mmHg)	–	74.6 (7.7)	75.5 (6.7)	0.670
				*df* = 24.1
				*p* = 0.510
				*η* ^2^ = 0.03

*M* men, *W* women, *BMI* body mass index, *HbA1c* hemoglobin glycated, *Tot chol* total cholesterol, *HDL chol* high-density lipoprotein cholesterol, *LDL chol* low-density lipoprotein cholesterol, *Trigl* triglycerides, *Microalb/creat* microalbuminuria/creatininuria, *SBP* systolic blood pressure, *DBP* diastolic blood pressure. ^a^ *t*–test applied after log-transformation. Statistical significance ^b^ *p* < 0.05 versus C; ^c^ *p* < 0.05 versus noDR

The difference among the three groups in terms of RNFL thickness was significantly dependent on quadrant (*F*(6;132) = 2.315; *p* = 0.037). This interaction was due to a specific difference in RNFL-N thickness, where both DM1 groups showed a similar reduction versus C (−3.9 for noDR and −4.9 for NPDR), without any relevant difference between them (−1.0).

**Fig. 1:**
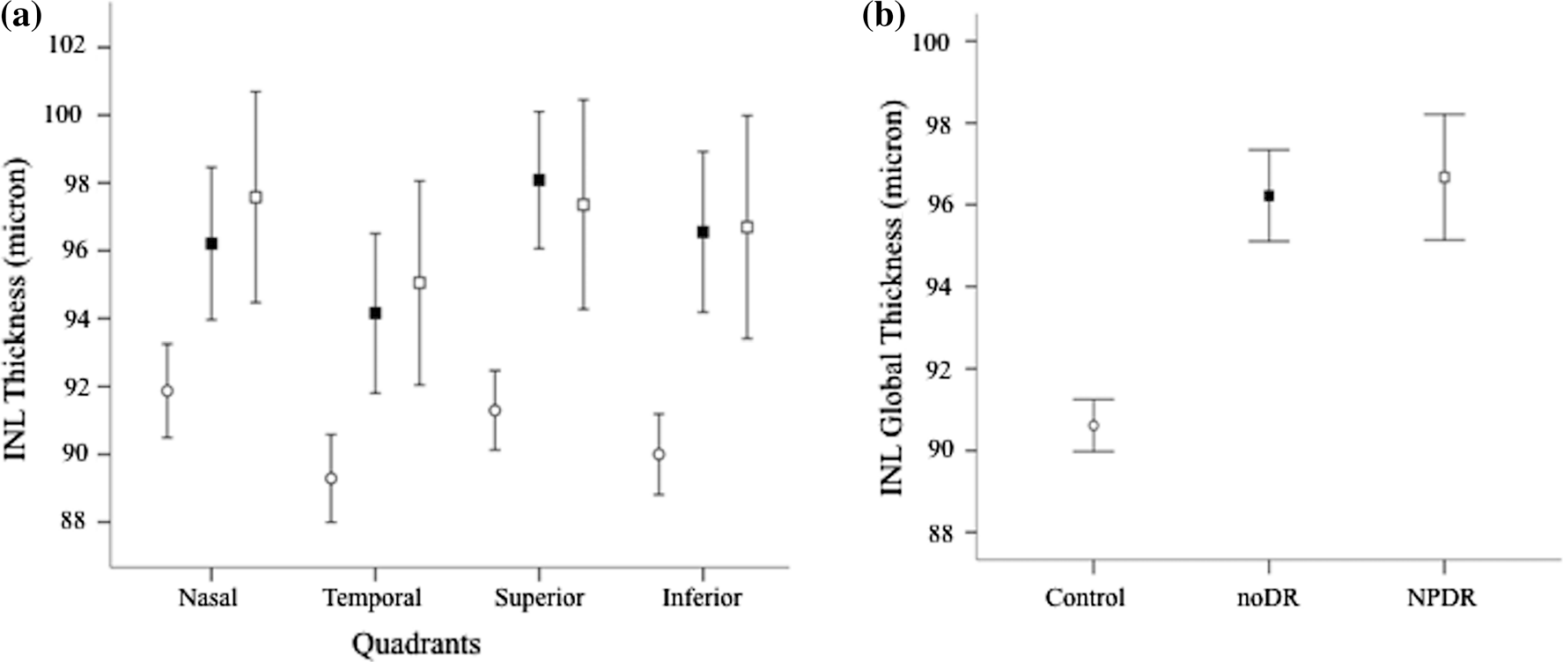
Thickness (mean ± SEM) of inner nuclear layer (INL) in the three groups: C (*open circle*), noDR (*closed square*) and NPDR (*open square*). Differences between groups were similar in each quadrant (**a**), allowing to obtain a global measure of INL (INL-G; **b**), indicating that both diabetic groups (noDR and NPDR) presented a significant increase (*p* < 0.01) in thickness versus C group

The lack of a significant interaction “Group X Sector” (*F*(3.4;75.1) = 1.112; *p* = 0.358) for INL thickness allows to consider a global measure of INL (INL-G), defined as the average value of the INL thickness of the four quadrants. Such value was significantly different in the three groups (*F*(2,44) = 3.468; *p* = 0.039), due to larger thickness in noDR group versus C (mean difference = 7.73; 95% CI: 0.32-15.14; *p* = 0.043), as well as in NPDR group versus C (mean difference = 7.74; 95% CI: 0.33–15.15; *p* = 0.043). On the other hand, INL-G thickness was very similar in the noDR and NPDR groups (*p* = 0.997; Fig. 1).

Since age, BMI and HDL chol were significantly different among the three groups, we added these variables as covariates in the previous analyses and the patterns remained stable.

Finally, the percentage of patients with both increase in INL-G thickness and decrease in RNFL-N thickness versus the average values of C group is 53% for noDR group and 28% for NPDR group.

### Correlation analysis

**Fig. 2:**
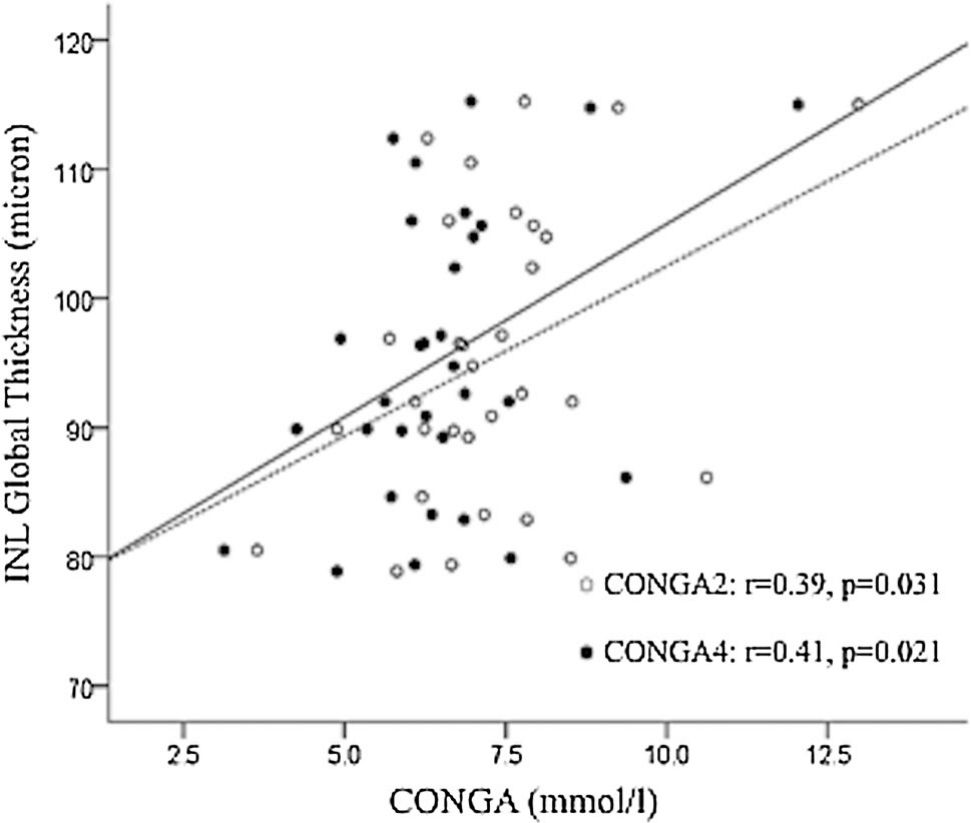
Scatter plot between CONGA-2, -4 and INL global thickness in Type 1 DM patients. CONGA-2 (*white circles*), CONGA-4 (*black circles*)

We did not observe significant correlation between HbA1c and retinal macular layers thickness (consistently, *p* > 0.2). In Type 1 DM patients, a negative correlation was observed between LBGI and RNFL-N quadrant (*r* = −0.382, *p* = 0.034); a positive correlation was observed between CONGA-1, -2 and -4 and INL-G (*r* = 0.40, *p* = 0.025; *r* = 0.39, *p* = 0.031; *r* = 0.41, *p* = 0.021, respectively; Fig. 2). In Type 1 DM patients, triglycerides were positively and significantly correlated to INL-G (*r* = 0.48, *p* = 0.011). Such correlation indexes were not significantly different in noDR and NPDR groups (consistently, *p* > 0.20).

### Multi-variable analysis

Because our data showed a significant effect of CONGA-1, -2, -4 and triglycerides on INL-G thickness, we assessed their effects through multivariable regression analysis. GV and triglycerides resulted both independent predictors of increased INL-G thickness. More precisely, 39% of INL variance resulted accounted for triglycerides (21%) and GV (18%; Triglycerides: slope 12.127; standard error 3.654; *t* 3.319; *p* value 0.003; cumulative *R* ^2^ 0.21; CONGA-2 slope 2.892; standard error 1.005; *t* 2.878; *p*-value 0.008; cumulative *R* ^2^ 0.39).

## Discussion

In our study, we observed an increased thickness of INL in persons with noDR and NPDR, compared to healthy groups. Our findings are in agreement with Vujosevic et al. [19] and more recently with Scarinci et al. [20]. Moreover, Bandello F et al. [21] reported that the increase in retinal thickness occurring in macular edema was predominantly located in the INL and extended to neighboring retinal layers in Type 2 DM patients. On the other hand, it has been recently showed a modest and selective reduction in the inner region of the INL in the temporal quadrant in Type 1 DM persons with no or mild RD [13], as well as in the pericentral area of macula in patients with minimal DR [22]. The subtle differences in the macular thickness described in these studies may be explained by the fact that the progressive phenomenon of retinal neurodegeneration was “photographed” in slightly different time phases, but with significantly different findings. In particular, INL includes mainly the nuclei of bipolar and Müller cells and the increase observed in this specific layer may represent a clinical sign of Müller cell activation [19]. Some recent studies have reported that Müller cells respond much earlier to high glucose conditions, than retinal vasculature [23]. Reactive gliosis represents the response of Müller cells to hyperglycemia [24], a process characterized by a specific pathophysiological mechanism: hypertrophy, cellular proliferation, and increased intermediate filament proteins nestin, vimentin and glial fibrillary acidic protein (GFAP) [25]. Increased levels of GFAP have been reported within 6–8 weeks of diabetes induction in animal models [26, 27]. Moreover, a recent study demonstrated that early Müller glial activation seems not to contribute to neurodegeneration, but might indeed have a neuroprotective activity against high glucose-induced neurotoxicity [28]. Therefore, reactive Müller cells are, at the beginning, neuroprotective, but consequently may stop supporting the neurons and contribute to neuronal degeneration [29].

On the other hand, the hypothesis of a vascular alteration to the basis of the increase in INL cannot be excluded.

Some studies [21, 30] showed in the early stages of subclinical macular edema, an increased retinal thickness, which mainly occurs at INL level, likely related to the pathological alteration of the deep capillary plexus and extracellular accumulation of fluid. The inner retina is provided mainly by the inner retina vasculature, which includes the superficial, intermediate and deep capillary plexus. Amacrine and horizontal cells have a specific connection with the intermediate and deep capillary plexus and at the same time interact with the Müller cells within INL. In the light of this particular anatomical structure of the inner retina, it is possible to speculate that the Müller cells have a crucial role in mediating relationship between retinal vessels and neurons [31–33] and, therefore, in the alteration of neurovascular unit that may occur in the early changes of DR, at the level of INL [20].

However, our observation of a significant increase in the INL thickness already in diabetic patients without RD compared to controls allows to hypothesize that the activation of Müller cells could represent the “*primum movens*” of the retinal neurodegeneration process.

In this study, we also observed a weakly decrease in RNFL thickness in the nasal quadrant of the macular area of diabetic eyes even no sign of DR or with very mild clinical signs of vascular retinopathy. Reduced RNFL thickness may be explained by progressive ganglion cells and astrocytes loss [19]. Different authors have reported a macular thinning of neuroretina suggesting that retinal neurodegeneration is an early event in diabetes mellitus, representing a preclinical stage of DR [34]. van Dijk et al. [22, 35] observed thinning of the total retina caused by a selective GCL thinning in the macular pericentral area and corresponding loss of RNFL thickness in the peripheral macula in patients with Type 1 DM with no or minimal signs of DR. In the pericentral area of the macula, the RNFL, GCL and IPL were thinner in persons with Type 2 DM and minimal DR compared to controls [32]. Vujosevic et al. [19] demonstrated a significant decrease in RNFL and at specific sites of GCL in the macula in Type 1 and 2 DM patients and no or mild RD. Moreover, Holm et al. [36] described that the nasal area of the macula, where there is a higher density of cones and ganglion cells, was more vulnerable to neurodegenerative processes than the temporal region, showing a lower amplitude and longer implicit time in this specific area with multifocal ERG analysis. In our study, we did not find significant difference in RNFL thickness between the diabetic groups without DR and with NPDR. This is probably due to the fact that all the patients with diabetes were at a very early stage of DR and in fairly good and comparable metabolic control.

In order to analyze the role of glycemic control on neuroretina, we performed a correlation analysis between overall glycemic load, expressed as HbA1c, and GV. It is noteworthy that only GV was associated with abnormalities of specific retinal layers, while no association was observed with HbA1c. In particular, we found a negative correlation between RNFL thickness and LBGI, that represents the frequency and the extent of low blood glucose excursions [18]. In vitro experiments showed a dose-dependent reduction in neuronal survival with decreasing concentrations of glucose in retinal cultures subjected to anoxia. Interestingly, the initial worsening of diabetic retinopathy observed in the early period of intensive glycemic control is manifested by the increase in the number of soft exudates, a typical feature of the ischemic retina. These findings indicate that glucose is a fundamental energy substrate in conditions of ischemia and that the ability of the retina to metabolically compensate during periods of low oxygen availability is impaired by low levels of blood glucose, although this is advantageous in terms of delaying the progression of diabetic retinopathy [37]. Therefore, our results confirm the hypothesis that the hypoglycemic excursions may be associated with retinal neurodegeneration [13]. Moreover, we also demonstrate that GV evaluated as CONGA, but not HbA1c, was positively associated with INL thickness, suggesting that an increase in intraday glycaemic variation seems to be involved in the development of an early activation of Müller cells. CONGA is unaffected by the asymmetry of a glycaemic profile, and it does not require identification of peak or nadirs according to arbitrary definitions, as opposed to the MAGE. Of particular interest, CONGA value under 3 h catches the smaller glycaemic swings (that occur over shorter time intervals), and intra-individual CONGA values increased with the time interval with a gradual leveling-off of values after an “*n*” of 3–4 h [38]. Therefore, our results support the concept that glial activation may be associated both with rapid and small glycemic peaks (expressed by CONGA-1 and- 2), and to the presence of more prolonged and “leveled” blood glucose excursions (CONGA-4). The pathophysiologic mechanism responsible for the association between GV and retinal thickness alterations could be represented by the activation of the oxidative stress pathway induced by GV [39], which is deleterious to the retinal nerve and glial cells [40]. In light of this results, the use of the new long-acting insulins, which are most effective in reducing the GV and hypoglycemia, may represent the best treatment choice in patients with Type 1 DM [41].

Finally, among all metabolic parameters, a positive association between triglycerides and INL thickness was observed. This association is independent of the GV effect on macular thickness. There is significant evidence from two major clinical trials (the FIELD and the ACCORD-Eye studies) [42, 43] that fenofibrate (FA), reducing triglycerides, arrests the progression of DR in Type 2 DM patients. The most accepted hypothesis is related to non-lipid mechanisms of FA. In particular, fenofibric acid exerts a protective effect on the microvasculature by suppressing apoptosis and stimulating endothelial nitric oxide synthase phosphorylation and nitric oxide production, which is mediated by 5′ adenosine monophosphate-activated protein kinase activation. FA, through the PPARα activation, reduces systemic inflammation, inhibits angiogenesis and neovascularization and increases plasma levels of adiponectin in hypertriglyceridemic patients, protecting against retinal vessel injury via modulation of tumor necrosis factor-*α* inflammatory responses. Finally, FA has protective effects on blood–retinal barrier breakdown [44]. Moreover, in a recent study, treatment with FA in mice with diabetes resulted in a significant decrease in both glial activation and the rate of apoptosis in GCL in comparison with mice treated with vehicle [45]. The authors did not associate these findings to the reduction in triglyceride levels. On the other hand, it has been described that apolipoprotein A1 (apo-A1), a key factor for the intraretinal lipids transport and a potent scavenger of reactive oxygen species, is overexpressed in all the neuroretinal layers in persons with diabetes [46]. These findings have led the authors to hypothesize that the higher content of apo-A1 during diabetes was a precocious protective mechanism; consequently, the tardive loss of capacity for apo-A1 production was associated to develop lipid deposition (hard exudates) and retinal damage induced by oxidative stress. To our knowledge, this is the first report showing a direct association between triglycerides levels and very early structural damage of neuroretina in Type 1 DM patients. Further studies are needed to understand the pathogenic mechanisms underlying such relationship.

Neuronal and glial cells interact with vascular cells in the retina forming a neurovascular unit, and Muller cells may represent the “communicator” cell between vessels and neurons [25]. The final balance of the reactive activation of Müller cells induced by glycemic excursions and triglycerides could be on the one hand the breakdown of endothelial cell–cell junctions, on the other hand, earlier, increased loss of retinal nerve fibers [47].

The novelty of our study lies in that the morphological alterations in retinal thickness were observed in a highly selected diabetic population, in good metabolic control and especially not affected by microvascular complications.

The main limitations of the present study were related to the small sample size, particularly of the controls. Moreover, CGM was performed only in Type 1 DM group, and therefore, the GV indices were available only for patients with diabetes and not for controls. The lack of follow-up data did not allow to analyze the prognostic role of GV and overall glycemic load on the onset and/or progression in neuroretinal abnormalities.

In conclusion, these results support the concept that glycemic excursions have an early neurodegenerative effect on the retina, which occurs even though the vascular component of DR is minimal. These findings could change our view on DR, traditionally regarded as a microvascular complication of diabetes, suggesting that DR could be a sensory neuropathy that affects the neuroretinal tissue [3]. A suggestive route to take would be to consider retinal neurodegeneration as a predictor of a neuropathic damage extended “beyond the retina” [48]. However, considerable evidence of a potential relationship between retinal neuropathy and peripheral or central nervous system damage is not still completely available.
